# Accuracy of Positioning and Risk Factors for Malpositioning Custom-Made Femoral Stems in Total Hip Arthroplasty—A Retrospective Multicenter Analysis

**DOI:** 10.3390/jpm13091285

**Published:** 2023-08-22

**Authors:** Philip Mark Anderson, Tizian Heinz, Dominik Rak, Jörg Arnholdt, Boris Michael Holzapfel, Silke Dorsch, Manuel Weißenberger, Rüdiger von Eisenhart-Rothe, Max Jaenisch, Max Ertl, Michael Wagner, Henning Windhagen, Maximilian Rudert, Axel Jakuscheit

**Affiliations:** 1 Department of Orthopedics, König-Ludwig-Haus, University of Würzburg, Brettreichstraße 11, 97070 Wuerzburg, Germany; 2 Department of Orthopedic Surgery, Klinikum Grosshadern, Ludwig-Maximilians-University of Munich, Marchioninistraße 15, 81377 Munich, Germany; 3 Department of Orthopaedics and Sports Orthopaedics, Klinikum Rechts der Isar, School of Medicine, Technical University of Munich, Ismaninger Straße 22, 81675 Munich, Germany; 4 Department of Orthopedics and Trauma Surgery, University Hospital Bonn, Venusberg Campus 1, 53127 Bonn, Germany; 5 Department of Orthopaedic Surgery, Klinikum Rechts der Isar, Technical University of Munich, Ismaninger Straße 22, 81675 Munich, Germany; max.ertl@mri.tum.de; 6 Department of Orthopaedics and Trauma Surgery, Klinikum Nürnberg Campus Süd, Paracelsus Medical University, Breslauer Straße 201, 90472 Nürnberg, Germany; michael.wagner@klinikum-nuernberg.de; 7 Department of Orthopaedic Surgery, Annastift Hannover, Medical School Hannover, Borries Str. 1-6, 30625 Hannover, Germany; henning.windhagen@diakovere.de

**Keywords:** total hip arthroplasty, custom-made implant, radiographs, hips

## Abstract

Total hip arthroplasty (THA) is commonly performed using off-the-shelf implants. In the case of a severe mismatch between the anatomy of the proximal femur and the geometry of the stem, the use of custom-made stems might become necessary. The goal of this study was to investigate the precision of the implantation of custom-made stems of one manufacturer (CTX stem, AQ Implants) and to determine risk factors for malpositioning. All patients receiving a custom-made CTX stem between 2014 and 2020 at six high-volume academic centers were retrospectively recruited. The achieved position of the stem, as determined by stem version, stem coronal angle, and implantation depth on radiographs, was compared to the plan. The influence of radiographic and demographic parameters on the position was investigated. The results revealed a high variability of the achieved implant position in relation to the preoperative plan. While the stem coronal angle only differed slightly from the intended position, the stem version and the implantation depth showed a high frequency and amount of deviation. Right stems showed significantly higher positions than planned. Surgeons must be aware of this potential problem when implanting custom-made stems.

## 1. Introduction

In light of the tremendous achievements made in this field during the 20th century, total hip arthroplasty was considered the “operation of the century” [1]. Today, total hip arthroplasty is one of the most frequently performed orthopedic operations worldwide, supported by strong evidence of the good results in terms of gain of quality of life and longevity of the implants, even in geriatric patients [2].

While the vast majority of these operations are performed with standard off-the-shelf implants of many different manufacturers, there are cases with strongly abnormal anatomy, making it impossible to use these standard implants mainly because of a mismatch between the shape of the implant and the proximal femur. These deformities can include alterations in all three plains leading to distortions of the version and the angularity of the proximal and diaphyseal femur [3]. Furthermore, often there are abnormalities of the width and the length of the femoral neck and the medullary cavity present, leading to a significant mismatch with standard implants, which are designed for the typical anatomy. This mismatch can lead to severe alterations in the biomechanics of the reconstructed hip, leg length discrepancies, and fractures of the femur and often prevents the stable fixation of cementless implants.

D’Antonio et al. presented a classification system of femoral abnormalities in total hip arthroplasty in 1993 [3]. This classification system summarizes six possible abnormalities: segmental bone defects, cavitary bone defects, combined bone defects, malalignment of the femur, femoral stenosis within the medullary canal, and femoral discontinuity.

The reasons for the distorted anatomy of the femur are either of innate or developmental nature or often a combination of both. The most frequent causal pathologies are hip dysplasia, previous femoral osteotomies, previous fractures of the femur, and Paget disease [4].

In these complex cases, either modular stem designs or custom-made stems have been proposed to achieve well-fixed implants and restore the physiological biomechanics of the hip [5]. Due to their extensive use in revision surgery, modular implants are usually easily available to address these complex cases. Furthermore, modular implants allow for intraoperative adjustments that might become necessary to achieve full stability and a free range of motion. However, modular implants have several potential disadvantages linked to modular junctions, e.g., corrosion, implant fracture, and release of metal ions. In particular, fractures of the neck and stem junction are a rather common devastating complication that might lead to extremely invasive revision surgeries.

To avoid such complications, custom implants might be used instead, but these implants are expensive and make preoperative computed tomography necessary [6,7]. Nevertheless, their use in cases of complex anatomy is supported by literature showing promising results with implant survival of 93–100% after 6–14 years [8]. While there is some evidence regarding the clinical results of these rarely used implants, there is a lack of evidence concerning their precise implantation, which is crucial for success. In order to close this gap of knowledge in the existing literature, this retrospective multicenter study was conducted to investigate the precision of the implantation of custom-made stems of one manufacturer (CTX stem, AQ Implants) in relation to the preoperative plan and to determine risk factors for malpositioning. Especially nowadays, where personalization is one of the most current topics in modern medicine, the use of custom-made implants is spreading as technical progress eases their availability and makes them more and more affordable. For this, it is important to gain more knowledge on the achievable results with these implants.

The investigated CTX individual endoprosthesis has been implanted since 1992. Since 1998, prosthesis manufacturing has been based on 3D data. The CTX individual endoprosthesis is made of a titanium alloy. It is proximally coated with hydroxyapatite, with the tip left uncoated. The CTX prosthesis is a short stem prosthesis, individually tailored based on computer tomography (CT) scans of the proximal femur and two X-ray images. The stem tapers distally to avoid contact with the diaphyseal cortex. The CTX prosthesis is intended to be implanted with metaphyseal press-fit with cortical bone contact desired only at the calcar, the lateral prosthetic shoulder, and the area of the trochanter minor. The goal of this fixation method is to ensure metaphyseal loading [9]. Planning and templating are 3D based and, therefore, likely to be more accurate than 2D planning [10].

## 2. Materials and Methods

After approval by the local ethics committee, all patients receiving a custom-made CTX stem between 2014 and 2020 at six high-volume academic centers were retrospectively recruited. Next to the implanted CTX stem, the only inclusion criterion was the availability of the pre- and postoperative records, including the preoperative CT scan with the planned position of the stem and the postoperative standardized X-rays revealing the achieved position. There were no exclusion criteria.

The protocol for the manufacturing of all CTX individual endoprostheses was based on CT scans of the proximal femur and additional slices through the knee condyles to determine the antetorsion. The physician marked the planned hip center and cup position. Based on this information, the proximal femur was 3D-reconstructed. The manufacturer used dedicated software to carry out virtual prosthetic planning and implantation into the 3D-reconstructed femur until stable cortical press-fit was achieved.

**Figure 1:**
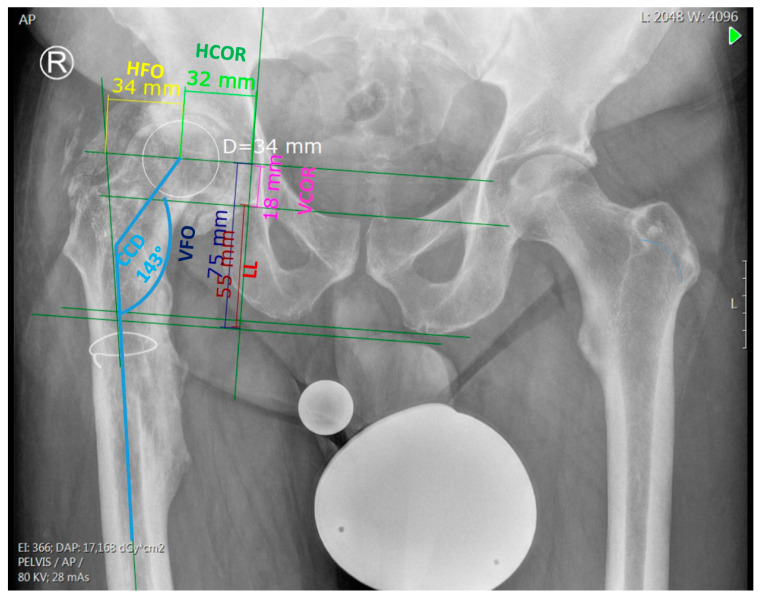
Preoperative measurements on AP pelvis X-ray. HFO: horizontal femoral offset. VFO: vertical femoral offset. HCOR: horizontal center of rotation. VCOR: vertical center of rotation. CCD: projected neck-to-shaft angle. LL: leg length.

After calibrating the pre- and postoperative X-rays (pelvis AP view) using the known diameter of the reference body or the implanted femoral head, the following radiographic landmarks and lines were determined (Figure 1):

- The center of the femoral head on the affected side.
- Both teardrops.
- Greater and lesser trochanter.
- A line tangent to the inferior border of both teardrops (inter-teardrop line).
- A line parallel to the inter-teardrop line passing through the center of the femoral head on the affected side.
- A line perpendicular to the inter-teardrop line crossing the deepest point of the teardrop (vertical teardrop line).
- A horizontal line connecting the ischial tuberosities.
- A horizontal line connecting the inferior borders of the lesser trochanter on both sides.
- The anatomical axis of the proximal femur.
- The longitudinal axis of the prosthetic neck.
- The longitudinal axis of the implanted stem.

Using these lines and landmarks, the following biomechanical parameters were measured on the preoperative X-ray using dedicated software (mediCAD, version 6.0, Hectec GmbH, Altdorf, Germany) (Figure 1):

- Horizontal femoral offset (HFO):
  The horizontal femoral offset is defined as the distance from the center of rotation of the femoral head to the anatomical axis of the proximal femur.
- Vertical femoral offset (VFO):
  The vertical femoral offset is defined as the distance from the center of rotation of the femoral head to the lower margin of the lesser trochanter.
- Horizontal center of rotation (HCOR):
  The horizontal center of rotation is defined as the distance from the center of rotation of the femoral head to the vertical teardrop line.
- Vertical center of rotation (VCOR):
  The vertical center of rotation is defined as the distance from the center of rotation of the femoral head to the inter-teardrop line.
- Projected neck-to-shaft angle (CCD):
  The projected neck-to-shaft angle is defined as the projected angle between the anatomical axis of the femur and the axis of the femoral neck.
- Leg length discrepancy (LLD):
  The leg length discrepancy is defined as the difference in the distance between the inferior margin of the lesser trochanter and the inter-teardrop line.

**Figure 2:**
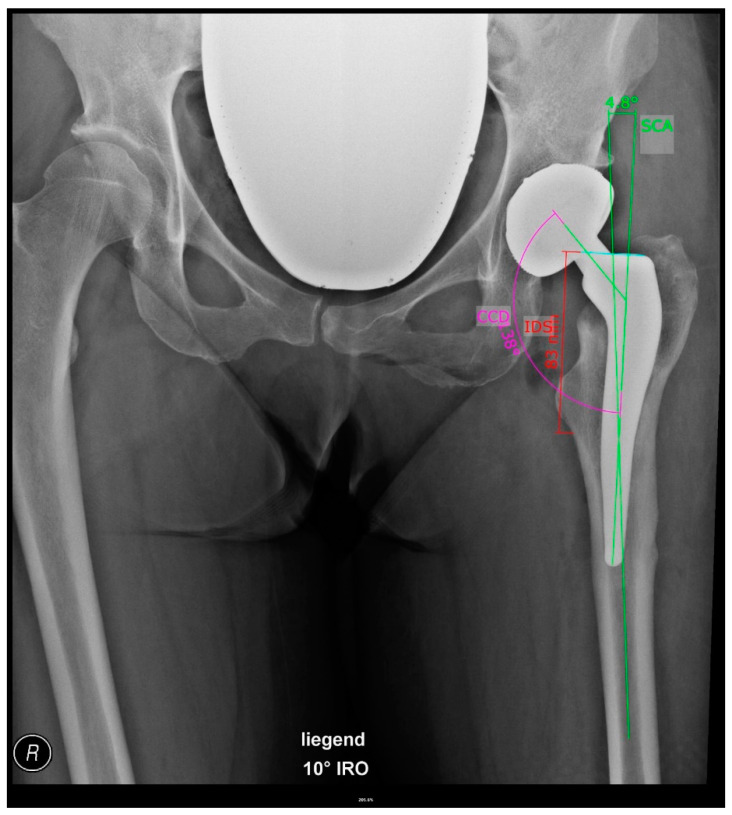
Postoperative measurement on AP pelvis X-ray in 10° internal rotation (IRO). Implantation depth of the stem (IDS), projected neck-to-shaft angle (CCD), and stem coronal angle (SCA).

The following parameters were measured on the postoperative X-ray to determine the achieved stem position (Figure 2):

- Stem version:
  The stem version was calculated by the formula presented by Weber et al. [11]:

Stem version = arcus ((tan (projected neck-to-shaft angle)/tan (real neck-shaft-angle))

- Stem coronal angle:
  The stem coronal angle is defined as the angle between the longitudinal axis of the implanted stem and the anatomical axis of the proximal femur.
- Implantation depth of the stem:
  The implantation depth of the stem is defined as the distance from the lower margin of the lesser trochanter to the shoulder of the stem.

The following information concerning the preoperative CT-based planning was recorded:

- Planned stem version;
- Planned stem coronal angle;
- Planned implantation depth of the stem.

Additionally, the following patient-related information was gathered:

- Body mass index (BMI);
- Gender;
- Age at time of surgery;
- Side operated on;
- Dorr classification of the morphology of the proximal femur [11];
- Surgical approach to the hip;
- Staging of osteoarthritis by the classification of Kellgren and Lawrence [12].

The primary outcome was the position of the implanted stem compared to the preoperative planning. The position was defined and compared by the implantation depth of the stem, the stem version, and the stem coronal angle.

Secondary outcome parameters were the influence of the patient-related factors and the biomechanical parameters on the achieved position.

Statistical analysis was performed using SPSS Statistics version 27 (IBM Inc., Armonk, NY, USA). Categorial data are presented as rates. Numerical data are presented as mean ± standard deviation. Group comparison of normal distributed numerical data was performed by *t*-test. Linear regression models were used to reveal the influence of the preoperative biomechanical and patient-related factors on the achieved position of the implanted stem. The level of significance was set at *p* < 0.05.

The histograms were designed with SigmaPlot for Windows, version 13.0 (Systat Software Inc. San Jose, CA, USA).

## 3. Results

The complete records of 41 hips in 38 patients were available. Of these, 31 were female (76%). The mean age at the time of surgery was 50 (±14) years and the mean BMI was 27.9 (±5.4) kg/m^2^. In 17 (41.5%) patients, the right side was operated on. In 15 (37%) cases, the anterior approach was chosen; in 10 (24%), the anterolateral; and in 16 (39%), the lateral. Table 1 gives an overview of the radiographic measurements on the preoperative X-rays that were performed on all 41 hips.

**Figure 3:**
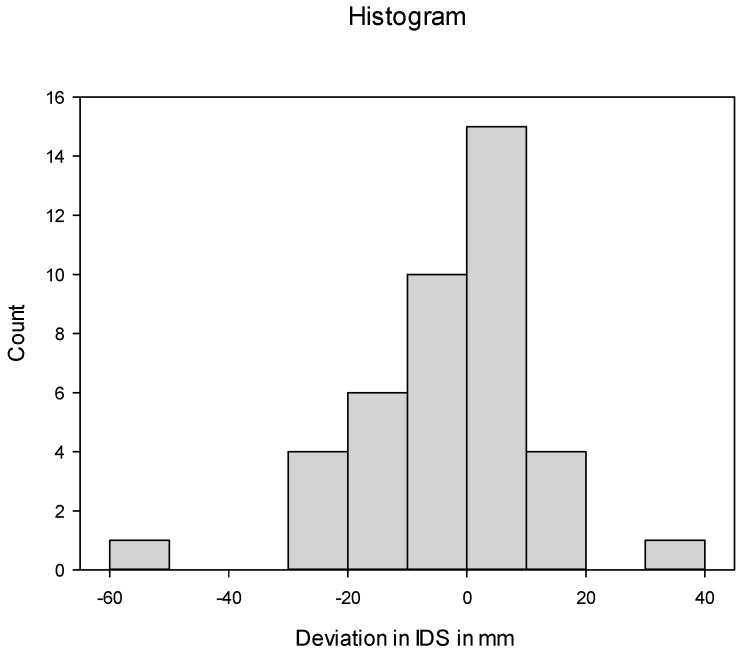
Deviation in postoperative stem implantation depth (IDS) from targeted IDS.

The stems were implanted on average 4.4 mm (±2.4 mm) less deep than planned. Only 15 (36.6%) stems were implanted within ±5 mm. A total of 17 (41.5%) stems were implanted more than 5 mm less deep than planned. In 18 patients (43.9%), the implantation depth of the stem differed by 10 mm or more from the plan (Figure 3).

**Figure 4:**
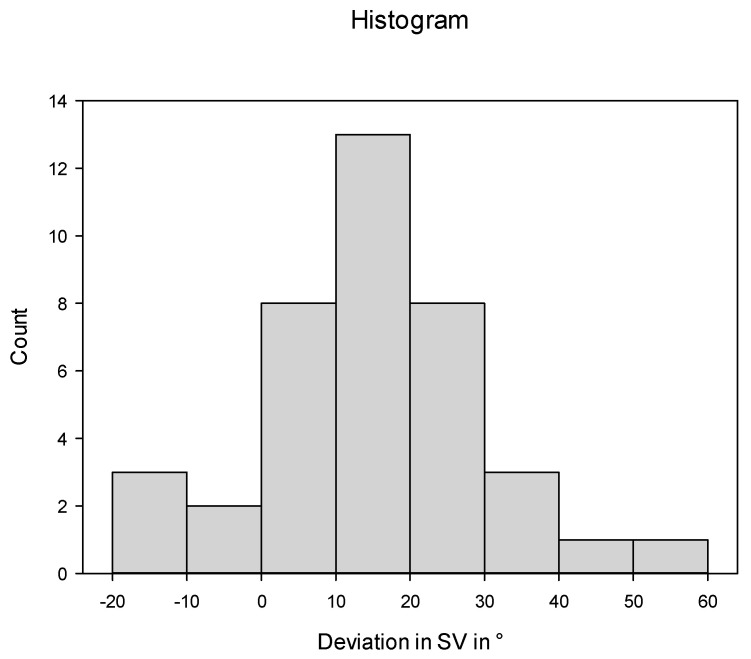
Deviation in the postoperative stem version (SV) from targeted SV.

The stems were implanted on average in 15.3° (±15.6°) more antetorsion than planned. Only three (7.3%) stems were implanted within ±5°. In 29 (70.7%) patients, the stem version differed 10° or more from the plan (Figure 4).

**Figure 5:**
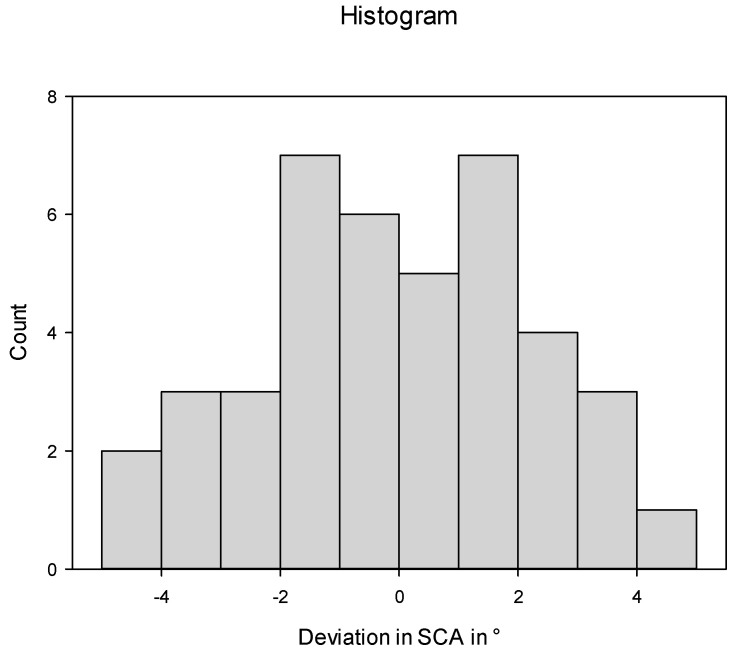
Deviation in postoperative stem coronal angle (SCA) from targeted SCA.

The stems were implanted on average in 0.1° (±2.3°) more valgus than planned. In all stems (100%), the stem coronal angle was within ±5° (Figure 5).

Only four (9.8%) stems were implanted within ±5° stem version, ±5 mm implantation depth, and ±5° stem coronal angle. Eight (19.8%) stems were implanted within ±10° stem version, ±10 mm implantation depth, and ±10° stem coronal angle.

None of the three parameters defining the position of the stem differed significantly between minimally invasive muscle-sparing approaches and the transgluteal lateral approach (Table 2).

Regression analysis was performed to study the influence of the biomechanical parameters and the patient’s characteristics on the achieved position.

A short distance between the inferior margin of the lesser trochanter and the inter-teardrop line was a predictor for increased antetorsion of the stem (*p* = 0.03). The right side was strongly associated with a less deep position of the stem (*p* = 0.01). In relation to the planned position, stems on the right side were implanted 12.4 mm (±19.1 mm) less deep, whereas stems on the left side were implanted 1.2 (±8.6) mm deeper than planned.

## 4. Discussion

The most important finding of this study is the high variability of the achieved implant position in relation to the preoperative plan. Not even 10% of the stems showed a position within a commonly accepted range of ±5° of stem version and stem coronal angle and ±5 mm of implantation depth. While the stem coronal angle only differed slightly from the intended position, the stem version and the implantation depth showed a high frequency and amount of deviation from the plan.

Especially, the fact that over 40% of the stems were implanted more than 5 mm less deep than intended raises concerns about clinical disadvantages for the patient as this will often lengthen the leg in a substantial manner. Even though lengthening of the leg is common after total hip arthroplasty, it is known to be a potential source of persistent pain and functional impairment and therefore is the most common reason for litigation against the surgeon [13]. Within the orthopedic literature, the incidence of substantial leg length discrepancy reported varies between 1% and 27%, whereby lengthening of the operated leg is far more frequent than shortening [14]. It remains uncertain where the boundaries between asymptomatic and symptomatic leg length differences lie. Some authors even postulated that there was no association between leg length discrepancy on the one side and patient satisfaction and functional outcome on the other side at all [15]. Despite these controversies, within most orthopedic communities, it seems to be a consensus that 10 mm differences are usually well tolerated by the patient. When applying this boundary, there remains 12% of patients in the investigated cohort in which the operated leg was lengthened substantially due to insufficient deep implantation of the stem.

The only predictive factor for higher stem position was the implantation in right hips, whereas left hips tended to have deeper implantation than planned. The reason for this finding remains unclear. A possible explanation might be the fact that approximately 90% of the population are right-handed and that this can be assumed to be true for orthopedic surgeons too, even though clear evidence on this is lacking [16,17]. While implanting a stem in a left hip in the supine position can be easily performed with the mallet in the right hand, implantation in a right hip makes either the use of the weaker left hand or a backhand technique necessary, both possibly preventing the surgeon from achieving the necessary amount of force. Pennington et al. published their investigation on implant position in total hip arthroplasty in left- and right-handed surgeons [18]. In contrast to the position of the acetabular component, they could not find a significant effect on the stem position. Mehta et al. published their investigation on the influence of handedness in total knee arthroplasty and were able to show that a right-handed surgeon produced less favorable results when implanting knee prostheses in left knees while changing the side of the table he stood on according to the side operated on [19]. The odds of having a poor outcome were nearly 1.4 times higher on the left side. While not proven, a relation to differences in dexterity and proprioception on the non-dominant side was assumed. In a similar manner, Molony et al. investigated the influence of handedness on malpositioning and consecutive failure of sliding hip screws in internal fixation of proximal femur fractures [20]. They reported a significantly higher risk in left hips when the surgeon was right-handed. In summary, although not well investigated, the available research points to handedness as a relevant factor in joint surgery.

Minimally invasive approaches during total hip arthroplasty are often deemed technically demanding, especially because of the limited exposition of the proximal femur. Spaans et al., for example, reported twice as long operating time, twice as much blood loss, and elevated early complication rates in the minimally invasive anterior approach compared to the postero-lateral approach without learning effect after 46 patients [21]. Martin et al. published the results of a prospective randomized controlled trial on the minimally invasive antero-lateral approach compared to a standard lateral approach [22]. They, too, found a significantly increased operating time and encountered several challenges in exposing the proximal femur. Despite these well-described technical difficulties in minimally invasive approaches to the hip, there was no significant difference in the achieved stem position between those stems implanted via the minimally invasive anterior or antero-lateral approach and those implanted via the standard lateral approach. This is in accordance with a systematic review by Den Hartog et al. showing no difference in the position of the components between the anterior approach and others [23]. Innmann et al. investigated the position of the cup and the stem between a minimally invasive anterolateral and a standard lateral approach [24]. They also could not find any influence on the stem position, whereas the risk of malpositioning the cup was higher in patients operated on via the anterolateral approach.

The observed high deviation in the stem version might lead to impingement of the prosthesis neck at the acetabular component, possibly causing reduced range of motion, instability, and increased wear, whereby the combined functional anteversion as determined by the stem version and the functional version of the acetabular component must be taken into account [25]. An increasing number of studies are highlighting the importance of spino-pelvic alignment in total hip arthroplasty, as the position of the acetabular component is highly variable throughout different movements and between different individuals with their variable spino-pelvic relationships [26]. Therefore, static parameters like the traditional “Lewinnek safe zone” are not reliable for determining if the combined positions of the stem and the cup will work together or lead to mechanical conflicts [27].

The only predictive factor for an increased antetorsion of the stem was a short distance between the inferior margin of the lesser trochanter and the inter-teardrop line on the preoperative radiograph. The reason for this remains unclear. To the best of our knowledge, there has been no published study describing this correlation before. A short distance between the inferior margin of the lesser trochanter and the inter-teardrop line refers to hips with short varus femoral necks with low neck-to-shaft angles. This anatomic variant has been identified as a risk factor for coronal malalignment in former studies [28,29]. The possible influence on the stem version was not investigated. A possible explanation might be the reduced working space in these hips leading to a limited exposition of the proximal femur. Regardless of the explanation of this finding, literature reports a high variability and bad predictability of the stem version in cementless off-the-shelf stems with a trend to increased anteversion [30,31,32,33]. Our results support the thesis that this is true for custom-made stems, too.

To our knowledge, the only other study investigating the accuracy of implantation of custom-made CTX stems was published by Rittmeister et al. in 2004 [34]. Within 107 hips, they found 31 (29%) stems implanted more than 5 mm less deep than intended—similar to our results. Within their cohort, they were able to show that dysplasia of the hip was associated with a less deep stem position. Furthermore, the position was dependent on the operating surgeon. In contrast to our findings, the side showed no effect on the stem position.

Independent of the accuracy of the achieved stem position, custom-made stems show good results throughout orthopedic literature. Jacquet et al. published their results on the use of customized stems in patients aged 50 years or less and patients suffering from developmental dysplasia of the hip. When taking aseptic loosening with consecutive revision as an endpoint, the survival rates were 96.8% and 96.81%, respectively. Good functional outcomes measured by the Harris Hip Score and the Merle D’Aubigne–Postel score were achieved in these demanding patient populations [35]. Pakos et al. published long-term results of 67 patients with congenital hip disease who had undergone total hip arthroplasty with the Symbiose custom-made femoral stem. At 10 years follow-up, the overall survival of the femoral component was 97.6%, and the survival related to aseptic loosening was 100% [36].

When interpreting the results of this study, one must be aware of its limitations, caused mainly by the retrospective design and the small number of included patients. Even though six high-volume academic centers reported their cases over a period of six years, only 41 hips could be recruited, highlighting the problem of achieving sufficient case numbers in studies on these rarely used implants. Furthermore, even though only implants of one manufacturer were included, every custom-made stem is unique by definition and therefore adds an additional possible confounder to the results, limiting the transferability to custom-made stems of other manufacturers.

During implantation, no navigation tool or robotic device was used. Current literature mostly shows the benefit of these techniques in terms of achieving the planned position of the cup and restoring the biomechanics of the hip [37,38,39]. Concerning the stem, there is conflicting evidence on whether the planned position can be achieved more accurately with navigation tools or robots. Koper et al. in 2019 published their multicenter randomized controlled trial on the value of navigation in hip resurfacing using the BrainLab Ci™ ASR System 1.0 (BrainLAB AG, Feldkirchen, Germany) [40]. Compared to conventional implantation, there was no significant difference in the accuracy of the placement of the femoral component. Wang et al. recently published their meta-analysis on radiological and clinical outcomes between robot-assisted and conventional total hip arthroplasty [38]. With the use of robots, the desired coronal alignment of the stem was achieved significantly better.

In light of the recent literature, it can be postulated that additional technical support by means of navigation tools or robots can reduce the risk of malpositioning the stem and—if used—might have changed the presented results significantly.

Neither the position of the acetabular component nor clinical or radiographic follow-up examinations were included in this study. Consequently, the clinical impact of the deviation in the stem version and the implantation depth of the stem cannot be determined.

Furthermore, the limitations of measuring the stem version on radiographs must be acknowledged even though the method used has shown high accuracy and high correlation compared to CT-based measurements [41].

Nevertheless, despite the mentioned limitations, this study adds important findings to the existing literature and might be of great interest to orthopedic surgeons dealing with these highly complicated cases.

## 5. Conclusions

Even though they are custom-made on the basis of individual anatomy, CTX stems are likely to show relevant deviation from the planned position. Especially in right hips, surgeons must be aware of the danger of implanting the stem substantially higher than planned, with possible negative effects on the leg length. Despite the preoperative plan and the custom-made nature of the implant, the achieved stem version often differs significantly from the planned position. Therefore, it might be helpful to carry out a trial reduction with trial implants in place in order to be able to adjust the position of the stem and or the cup in case of severe leg length discrepancy, impingement, or instability.

## Figures and Tables

**Table 1:** Preoperative radiographic measurements.

Measured Parameters	Mean	±SD
HFO (mm)	26.4	±13.2
VFO (mm)	68.4	±19.9
HCOR (mm)	32.7	±11.0
VCOR (mm)	24.6	±9.3
CCD (°)	136	±22.4
LLD (mm)	9.4	±18.4
Kellgren and Lawrence Score	*n*	(%)
0	5	(12.2)
1	2	(4.9)
2	15	(36.6)
3	8	(19.5)
4	11	(26.8)
Dorr Classification Type	*n*	(%)
A	15	(36.6)
B	18	(43.9)
C	8	(19.5)

HFO—horizontal femoral offset; VFO—vertical femoral offset; HCOR—horizontal center of rotation; VCOR—vertical center of rotation; CCD—projected neck-to-shaft angle; LLD—leg length discrepancy.

**Table 2:** Deviations of postoperative stem positions from the targeted position under minimally invasive muscle-sparing approaches and the transgluteal lateral approach.

	Minimally Invasive Anterior and Antero-Lateral Approach (*n* = 25)		Lateral Approach (*n* = 16)		*p*
Stem version deviation in ° (mean ± SD)	18.0	±17.9	11.4	±10.9	0.198
Stem implantation depth deviation in mm (mean ±SD)	−5.3	±17.8	−3.1	±10.8	0.657
Stem coronal angle deviation in ° (mean ± SD)	0.1	±2.2	−0.4	±2.4	0.516

*n*—number of hips; SD—standard deviation; *p*—significance.

## Data Availability

The data presented in this study are available on request from the corresponding author. The data are not publicly available due to privacy policy.
